# Systemic Administration of a Brain Permeable Cdk5 Inhibitor Alters Neurobehavior

**DOI:** 10.3389/fphar.2022.863762

**Published:** 2022-05-12

**Authors:** Alan Umfress, Sarbjit Singh, Kevin J. Ryan, Ayanabha Chakraborti, Florian Plattner, Yogesh Sonawane, Jayapal Reddy Mallareddy, Edward P. Acosta, Amarnath Natarajan, James A. Bibb

**Affiliations:** ^1^ Department of Surgery, University of Alabama at Birmingham, Birmingham, AL, United States; ^2^ Eppley Institute for Research in Cancer and Allied Diseases, University of Nebraska Medical Center, Omaha, NE, United States; ^3^ Department of Pharmacology and Toxicology, University of Alabama at Birmingham, Birmingham, AL, United States; ^4^ Neuro-Research Strategies, Houston, TX, United States; ^5^ Departments of Neurobiology and Neurology, University of Alabama at Birmingham, Birmingham, AL, United States; ^6^ O’Neil Comprehensive Cancer Center, University of Alabama at Birmingham, Birmingham, AL, United States

**Keywords:** cyclin-dependent kinase 5, pharmacokinetics, inhibitors, behavior, kinase inhibitors

## Abstract

Cyclin-dependent kinase 5 (Cdk5) is a crucial regulator of neuronal signal transduction. Cdk5 activity is implicated in various neuropsychiatric and neurodegenerative conditions such as stress, anxiety, depression, addiction, Alzheimer’s disease, and Parkinson’s disease. While constitutive Cdk5 knockout is perinatally lethal, conditional knockout mice display resilience to stress-induction, enhanced cognition, neuroprotection from stroke and head trauma, and ameliorated neurodegeneration. Thus, Cdk5 represents a prime target for treatment in a spectrum of neurological and neuropsychiatric conditions. While intracranial infusions or treatment of acutely dissected brain tissue with compounds that inhibit Cdk5 have allowed the study of kinase function and corroborated conditional knockout findings, potent brain-penetrant systemically deliverable Cdk5 inhibitors are extremely limited, and no Cdk5 inhibitor has been approved to treat any neuropsychiatric or degenerative diseases to date. Here, we screened aminopyrazole-based analogs as potential Cdk5 inhibitors and identified a novel analog, 25–106, as a uniquely brain-penetrant anti-Cdk5 drug. We characterize the pharmacokinetic and dynamic responses of 25–106 in mice and functionally validate the effects of Cdk5 inhibition on open field and tail-suspension behaviors. Altogether, 25–106 represents a promising preclinical Cdk5 inhibitor that can be systemically administered with significant potential as a neurological/neuropsychiatric therapeutic.

## Introduction

Cyclin-dependent kinase 5 (Cdk5) is a proline-directed kinase predominantly expressed in mature neurons (Dhavan and Tsai, 2001). Unlike traditional cyclin kinases, Cdk5 does not require coactivation by binding to a cyclin partner. Instead, Cdk5 is constitutively activated through interactions with its unique coactivators p35 or p39 (Dhavan and Tsai, 2001). Over 3 decades of research on Cdk5 functionality has revealed this kinase to play unique roles in modulating neuronal development, complex behavior, and neurodegeneration. Cdk5 is expressed within excitatory and inhibitory circuitry and regulates glutamatergic, dopaminergic, and GABAergic signaling (Liu et al., 2001; Chergui et al., 2004; Li et al., 2018). The current scenario shows that Cdk5 is a critical regulator of homeostatic neuronal signaling (Dhavan and Tsai, 2001; Barnett and Bibb, 2011; Plattner et al., 2014; Plattner et al., 2015). Cdk5 conditional knockout mice display reduced anxiety-like behaviors and enhanced cocaine locomotor sensitization (Bibb et al., 2001a; Meyer et al., 2008; Plattner et al., 2015). In addition, knockout of Cdk5 within the striatum of adult mice results in impaired long-term plasticity (LTP) and altered motor learning (Benavides and Bibb, 2004; Hernandez et al., 2016). Similarly, mice constitutively lacking the p35 co-factor have dramatically altered cortical layer architecture and display hyperactivity (Chae et al., 1997; Drerup et al., 2010). Within the hippocampus, Cdk5-dependent phosphorylation of NMDA receptors has been linked to decreases in the cell surface expression of NMDA receptors, thereby decreasing cell excitability. Infusion of Cdk5 targeting small-interfering peptides blocks Cdk5 phosphorylation of the NR2B (GluN2B) subunit of NMDA receptors, increases hippocampal LTP, and enhances fear-learning and memory (Hawasli et al., 2007; Plattner et al., 2014). Many other studies have assigned both pre-and post-synaptic functions for Cdk5 in virtually every brain region and associated circuitry. Together, these studies have established a crucial role for homeostatic Cdk5 signaling in normal brain function.

Conversely, Cdk5 activity also serves as a principle feature in excitotoxic signaling and has been implicated in Alzheimer’s disease (AD), Parkinson’s disease (PD), ischemia, and brain injury (Patrick et al., 1999; Meyer et al., 2014; Binukumar et al., 2015; Yousuf et al., 2016). This is because Cdk5 can transform from a constitutively active homeostatic kinase to an aberrantly active neurotoxic enzyme. For example, excitotoxicity or loss of membrane potential can cause loss of Ca^2+^ homeostasis. Increased intracellular Ca^2+^ can activate the protease calpain, which then cleaves Cdk5’s physiological coactivator p35 into a truncated aberrant coactivator p25 and a p10 fragment (Kusakawa et al., 2000). The p25 activator lacks a myristoylation site on the N-terminal region of p35. Consequently, the Cdk5/p25 complex is no longer membrane-sequestered (Dhavan and Tsai, 2001). Furthermore, p25 is degraded less quickly than p35 (Patrick et al., 1998). These combined factors lead to mislocalization and hyperactivation of the Cdk5 holoenzyme toward a subset of substrates, resulting in neuronal injury and cellular death (Dhavan and Tsai, 2001). For these reasons, Cdk5/p25 has been implicated in various neurogenerative diseases and neurotoxic insults (Patrick et al., 1999; Cruz et al., 2003; Miao et al., 2012; Sundaram et al., 2012). Elevated levels of the aberrant coactivator p25 induce AD pathologies, including cellular death and hyperphosphorylation of the neurofibrillary tangle protein, Tau (Cruz et al., 2003). Deletion or inhibition of the Cdk5/p25 complex is neuroprotective in various disease models (Sundaram et al., 2013; Yousuf et al., 2016). Thus, Cdk5 serves as a critical regulator of both homeostatic and pathological brain function, and inhibition of Cdk5 may be of significant therapeutic value.

Therapeutic infusion of preclinical inhibitors of Cdk5 into the brain has been successfully shown to ameliorate neurological disease phenotypes in mice. However, none of these compounds has proven effective in clinical trials (Cicenas et al., 2015; Pao and TSAI, 2021). A major barrier for most of these inhibitors has been specificity and tissue penetrance. Most CDK inhibitors target the ATP-binding site of the kinase domain. Unfortunately, cyclin-dependent kinase family members share high sequence homology, particularly in this drug-targeting domain (Sonawane et al., 2016), leading to nonspecific drug interactions and off-target inhibition (Khair et al., 2019). The most prominent Cdk5 inhibitor, roscovitine, has proven to be a useful tool in neuroscience and has also yielded promising results in the treatment of various cancers but has not been approved for use in neurological conditions (Cicenas et al., 2015). More recent work using small-interfering peptides (SiPs) that selectively block Cdk5-p25 but not Cdk5-p35 interactions by both pharmacological and transgenic means has shown promise in preclinical models of neurodegeneration and stroke (Binukumar et al., 2015; Binukumar and Pant, 2016). While these SiPs are proof of concept advances, the strategy of only targeting Cdk5/p25 limits this approach to excitotoxicity-/neuronal injury–linked disease rather than neuropsychiatric conditions mediated through Cdk5/p35 activity. Numerous CDK inhibitors have been developed for the treatment of various cancer types (Asghar et al., 2015; Mangini et al., 2015; Syed, 2017). Development of inhibitors with an aminopyrazole core that forms hydrogen bonds within the hinge region of kinases such as AT7519 has progressed through clinical trials. Derivation of aminopyrazole analogs has shown increased specificity for Cdk2/5 over other CDK family members and effectively halted preclinical models of cancer (Pevarello et al., 2004; Chen et al., 2014; Rana et al., 2018). However, the ability of these aminopyrazole analogs to penetrate the blood–brain barrier and effectively inhibit Cdk5 in the brain remains unknown.

Here, by screening a small panel of aminopyrazole analogs, we identify analog, 25–106, that can be delivered systemically, penetrate the blood–brain barrier, and pharmacologically inhibit Cdk5/p35 activity in the brain. These data demonstrate that 25–106 inhibits Cdk5 activity *in vivo* and displays high potency for inhibition *in vitro.* Furthermore, this inhibitor modulates behavioral phenotypes linked to Cdk5 through previous characterizations of conditional knockout mice. These studies further advance our understanding of Cdk5’s role in neuropsychiatric behavior, bring forth a new pharmacological tool to assess Cdk5 activity, and suggest a promising new preclinical drug.

## Methods

### Animals

The wild-type group housed male C57BL/6 mice 10–12 weeks of age were used for all *in vivo*, *ex vivo*, and behavioral studies. This age range was selected to be consistent with previous studies of conditional Cdk5 knockout mice (Plattner et al., 2015). 25–106 was administered in a 20-fold dose range intravenously (I.V.) at 10, 50, 100, and 200 mg/kg for pharmacokinetic studies. This delivery mode was used as I.V. injections to avoid first-pass drug metabolism observed using other injection methods (Pond and Tozer, 1984). The mice were subsequently euthanized 1, 2, 6, and 24 h post injection to monitor pharmacokinetic distribution and clearance rates from plasma, brain, liver, and kidney. All animal experiments were performed under approved protocols by the University of Alabama at Birmingham (UAB) Institutional Animal Care and Use Committees and in accordance with the guidelines of the Animal Welfare Act and the Guide for the Care and Use of Laboratory Animals.

### Synthesis of Cdk5 Inhibitors

All reagents were purchased from commercial sources and were used without further purification. Flash chromatography was carried out on silica gel (200–400 mesh). Thin-layer chromatography (TLC) was run on pre-coated ANALTECH uniplate and observed under UV light at 254 nM and with a basic potassium permanganate dip. Column chromatography was performed with silica gel (230–400 mesh, grade 60, Fisher Scientific, United States). 1H NMR (400 MHz) and 13C NMR (100 MHz) spectra were recorded in chloroform-d or DMSO-*d*6 on a Bruker-400 spectrometer. DMSO-*d*6 was 2.50 ppm for 1H and 39.55 ppm for 13C, and CDCl_3_ was 7.26 ppm for 1H and 77.23 ppm for 13C. Proton and carbon chemical shifts were reported in ppm relative to the signal from residual solvent proton and carbon. The data are presented as follows: chemical shift, multiplicity (s = singlet, d = doublet, t = triplet, q = quartet, p = pentet, m = multiplet and/or multiple resonances), coupling constant in hertz (Hz), and integration. The purity of the final compounds was determined by analytical HPLC and was found to be ≥95% pure. HPLC was performed on a Waters Alliance 2,690 system equipped with a Waters 2,996 photodiode array detector and an auto-sampler under the following conditions: column, Phenomenex Luna-2 RP-C18 (5 μM, 4.6 mm × 250 mm, 120 Å, Torrance, CA); solvent A, H_2_O containing 0.1% formic acid (FA); solvent B, CH_3_CN containing 0.1% FA; gradient, 60% B to 100% B over 8 min followed by 100% B over 6 min; injection volume, 25 μL; flow rate, 1 ml/min retention times and purity data for each target compound are provided in the Experimental Section. The purified compound was further confirmed by MS analysis using a Quattro Micro triple–quadrupole mass spectrometer using an electron spray ionization (ESI) technique and a TOF mass analyzer.

Synthesis of 2-(3,4,5-trimethoxyphenyl) acetyl chloride (Rana et al., 2018). To a solution of 3,4,5-trimethoxyphenylacetic acid (1 g, 4.42 mmol) in anhydrous dichloromethane at 0°C was added oxalyl chloride (1.7 g, 13.26 mmol), followed by the addition of the catalytic amount of dimethylformamide (DMF) (50 µL). The reaction mixture was allowed to warm to room temperature and stirred for 2 h. The completion of the reaction was monitored by TLC by quenching a small aliquot of the crude mixture in methanol. After completion of the reaction, the reaction mixture was concentrated to dryness, and the residue was used in the next step without further purification.

Synthesis of tert-butyl 3-amino-5-cyclobutyl-1H-pyrazole-1-carboxylate (2) (Rana et al., 2018). 3-amino-5-cyclobutyl-1H-pyrazole (1.86 g, 13.55 mmol) in dichloromethane was placed in a round-bottomed flask, and 4N KOH (6.10 g, 108.4 mmol) was added to the stirring solution. The reaction mixture was allowed to stir at room temperature, followed by the addition of Boc anhydride (3.11 g, 14.23 mmol) in small batches. The reaction mixture was monitored for completion of the reaction by TLC (∼3 h), and the mixture was diluted with CH_2_Cl_2_, washed with brine, and dried with MgSO_4_. The crude product was purified using silica gel column chromatography (2.76 g, 86%).

Synthesis of tert-butyl 5-cyclobutyl-3-[2-(3,4,5-trimethoxyphenyl)acetamido]-1H-pyrazole-1-carboxylate (3) (Rana et al., 2018). Compound 2 (tert-butyl 3-amino-5-cyclobutyl-1H-pyrazole-1-carboxylate, 257 mg, 1.08 mmol) was dissolved in dichloromethane followed by addition of diisopropylethylamine (280 mg, 2.16) under N_2_. The reaction mixture was cooled to 0°C and freshly prepared 2-(3,4,5-trimethoxyphenyl)acetyl chloride (345 mg, 1.40 mmol) dichloromethane was added dropwise at 0°C. Following addition, the reaction temperature was allowed to rise to room temperature and, the mixture was stirred for an additional 3 h. The crude mixture was washed with brine, extracted with dichloromethane, dried with MgSO_4_, and concentrated. The crude mixture was purified using silica gel column chromatography (381 mg, 79%); 1H NMR (400 MHz, CDCl_3_) *δ* 10.31 (s, 1H), 6.87 (s, 1H), 6.57 (s, 2H), 3.91 (s, 6H), 3.87 (d, J = 3.8 Hz, 3H), 3.72 (s, 2H), 3.55 (m, 1H), 2.41–2.14 (m, 4H), 2.12–1.85 (m, 2H), 1.72–1.56 (m, 9H).

Synthesis of N-(5-cyclobutyl-1H-pyrazol-3-yl)-2-(3,4,5-trimethoxyphenyl)acetamide (25–106). To a stirred solution of tert-butyl 5-cyclobutyl-3-[2-(3,4,5-trimethoxyphenyl)acetamido]-1H-pyrazole-1-carboxylate (3, 60 mg) in dichloromethane (5 ml) at 0°C, trifluoroacetic acid (1 ml) was added dropwise, and the reaction mixture was stirred at room temperature for 3 h. After completion, the reaction mixture was concentrated *in vacuo*, and the resulting solid was recrystallized to yield a colorless solid (35 mg, 76%). Purity was assessed by HPLC (>98%); 1H NMR (400 MHz, DMSO) δ 12.04 (s, 1H), 10.41 (s, 1H), 6.64 (s, 2H), 6.31 (s, 1H), 3.76 (s, 6H), 3.63 (s, 3H), 3.51 (s, 2H), 3.48–3.37 (m, 1H), 2.35–2.18 (m, 2H), 2.18–2.02 (m, 2H), 1.95 (m, 8.6 Hz, 1H), 1.89–1.73 (m, 1H); 13C NMR (100 MHz, DMSO) *δ* 168.1, 153.1, 147.8, 147.5, 136.7, 132.2, 107.0, 93.8, 60.4, 56.3, 43.3, 31.6, 29.4, 18.6; MS calculated for C_18_H_23_N_3_O_4_ m/z 345.16, found mass: 346.14.

### LC-MS/MS Detection of 25–106

A quantitative determination of 25–106 in mouse plasma and tissue homogenate was accomplished by the use of protein precipitation and high-performance liquid chromatography with tandem mass spectrometry detection (LC-MS/MS). 5-(N,N-hexamethylene) amiloride (HMA) was used as the internal standard (IS). 25–106 and IS were typically extracted from 50 µL of mouse plasma or tissue homogenate using protein precipitation with methanol. The extracts were analyzed by reverse-phase chromatography using an ACE Excel 3 C18-PFP column under isocratic conditions at a flow rate of 500 μL/min. The column temperature was maintained at 40°C. The mobile phase A consisted of 0.1% formic acid in water, and the mobile phase B was acetonitrile. A triple–quadrupole mass spectrometer (AB Sciex 5,000) equipped with TurboV IonSpray operating in positive-ion mode was used. Column effluents were analyzed by multiple reaction monitoring (MRM). The precursor/product transitions are 346.1 to 137.1 m/z for 25–106 and 312.37 to 129.5 m/z for IS. The calibration curve was fit using weighted (1/x^2^) linear regression analysis of the 25–106/IS peak area ratio vs. the 25–106 concentration from 1.0–80.0 ng/ml (ng/g for tissue) for the low curve and 80.0–10,000 ng/ml (ng/g for tissue) for the high curve. The concentrations of incurred and quality control samples were calculated with the same regression analysis, and the results were reported in ng/mL (plasma) or ng/g (tissue) of 25–106.

### Molecular Modeling and Docking Analysis

Molecular modeling was performed using the Schrödinger small-molecule drug discovery suite 2020–1. The CDK/p25 (PDB: 1H4L) crystal structure was retrieved from the Protein Data Bank and analyzed using Maestro version 12.3.013 (Schrödinger Inc.). The protein preparation was performed using Protein Preparation Wizard in which missing hydrogen atoms, side chains, and loops were added, followed by grid generation, ligand preparation, and docking. The protein structures were minimized using the OPLS3e force field to optimize hydrogen bonding networks and converging heavy atoms to an RMSD of 0.3 Å. The prepared protein structure was subjected to SiteMap for binding site analysis as implemented in Schrödinger Suite 2020–1. At least 15 site points per reported site were required. A more restricted definition of hydrophobicity together with a standard grid was used. Site maps at 4 Å or more from the nearest site points were cropped. The structures of 25–106 and roscovitine were subjected to Lig Prep to generate conformers and possible protonation at pH of 7 ± 2, which serves as an input for the docking process. The receptor grid was generated using the receptor grid generation tool in Maestro (Schrödinger Inc.). GLIDE XP was used to perform all the dockings with the van der Waals radii of nonpolar atoms for each of the ligands being scaled by a factor of 0.8 and the partial charge cutoff of 0.15. The docked poses from GLIDE XP were analyzed using Maestro version 12.3.013 (Schrödinger Inc.).

### *Ex Vivo* Acute Brain Slice Pharmacology

Brain slice pharmacology was performed as described (Nishi et al., 2000). Briefly, the mice were decapitated, and the brains were rapidly dissected and submerged in ice-cold normal Kreb’s solution (124 mM NaCl, 4 mM KCl, 26 mM NaHCO_3_, 1.25 mM KH_2_PO_4_, 1.5 mM MgSO_4_, 10 mM d-glucose, and 1.5 mM CaCl_2_). The brains were subsequently sectioned into normal Kreb’s solution at 350 µ thickness. The brain slices were transferred to a 30°C solution of normal Kreb’s containing 10 µM deaminase for a 1-h recovery period. The sections were then incubated in normal Kreb’s solution with and without the indicated compounds across the dose and time ranges indicated. The sections were rapidly snap-frozen in dry ice to terminate treatment regimes.

### Immunoblotting

Immunoblotting was performed as previously described (Umfress et al., 2021). Mouse brains were rapidly dissected in an ice-cold solution of 50 mM NaF/PBS. The regions of interest were further dissected out and rapidly snap-frozen in dry ice. The frozen tissue was homogenized in boiling lysis buffer (1% SDS, 50 mM NaF) and sonicated with several three to four pulses at 40 dB. The samples were subsequently denatured at 90°C for 5 min. Protein concentration was determined by the BCA assay, and homogenates were diluted in 4x sample buffer, separated by SDS-PAGE, and transferred to 0.45-µm nitrocellulose membranes, blocked with 5% milk solution (1 h), and probed with primary antibodies (overnight). The following antibodies were used in the study: phospho-Ser549 synapsin I (Phosphosolutions), total synapsin I (Phosphosolutions), actin (Invitrogen), phospho-Ser1116 (in-house) (Plattner et al., 2014), and total NR2B (Phosphosolutions), phospho-Thr75, and total DARPP-32 (Cell Signaling Technology). Visualization was performed using the Licor CLX membrane scanner and Licor secondary antibodies.

### *In Vitro* Kinase Assays

*In vitro* phosphorylation reactions were conducted as dose-response kinase inhibition assays with three highly homologous CDKs, namely, Cdk2/CyclinE (1.5 nM), CDK5/p25 (0.35 nM), and Cdk9/CyclinT1 (9 nM). Briefly, the abovementioned kinases were incubated with ATP (30 μM), the corresponding substrate (20 μM), and an increasing concentration of 25–106 (0–5 μM) for 30 min. The kinase activity of the abovementioned reactions was graphed against the concentration of 25–106 to determine the IC_50_ and K_i_ values for each kinase.

### Neurobehavior

#### Open-Field Test

The OFT was performed as described (Chakraborti et al., 2021). With the adaptation duration of the test being 1 h for each mouse, briefly, the mice were placed in a square box (40 cm × 40 cm) and allowed to freely explore. The center square portion of the maze was defined as the “open area.” Video tracking using EthoVision 16 software was used to track animal movement throughout the box and stereotypic and locomotive behaviors such as hopping, sniffing, and rearing. Total distance traveled, entries into the center of the maze, and duration in the center were tracked and analyzed in EthoVision.

#### Tail Suspension Test

The tail suspension test was conducted as previously described (Plattner et al., 2015). The mice were suspended by their tails for a total duration of 6 min and video-recorded. Post-hoc blind experimentalists quantified the duration and latency of immobility for each randomized video. The average quantitative determents of each observer were averaged to generate the final analytic values of motion.

## Results

### Synthesis of Aminopyrazole Analogs as Novel Cyclin-Dependent Kinase 5 Inhibitors

**FIGURE 1:**
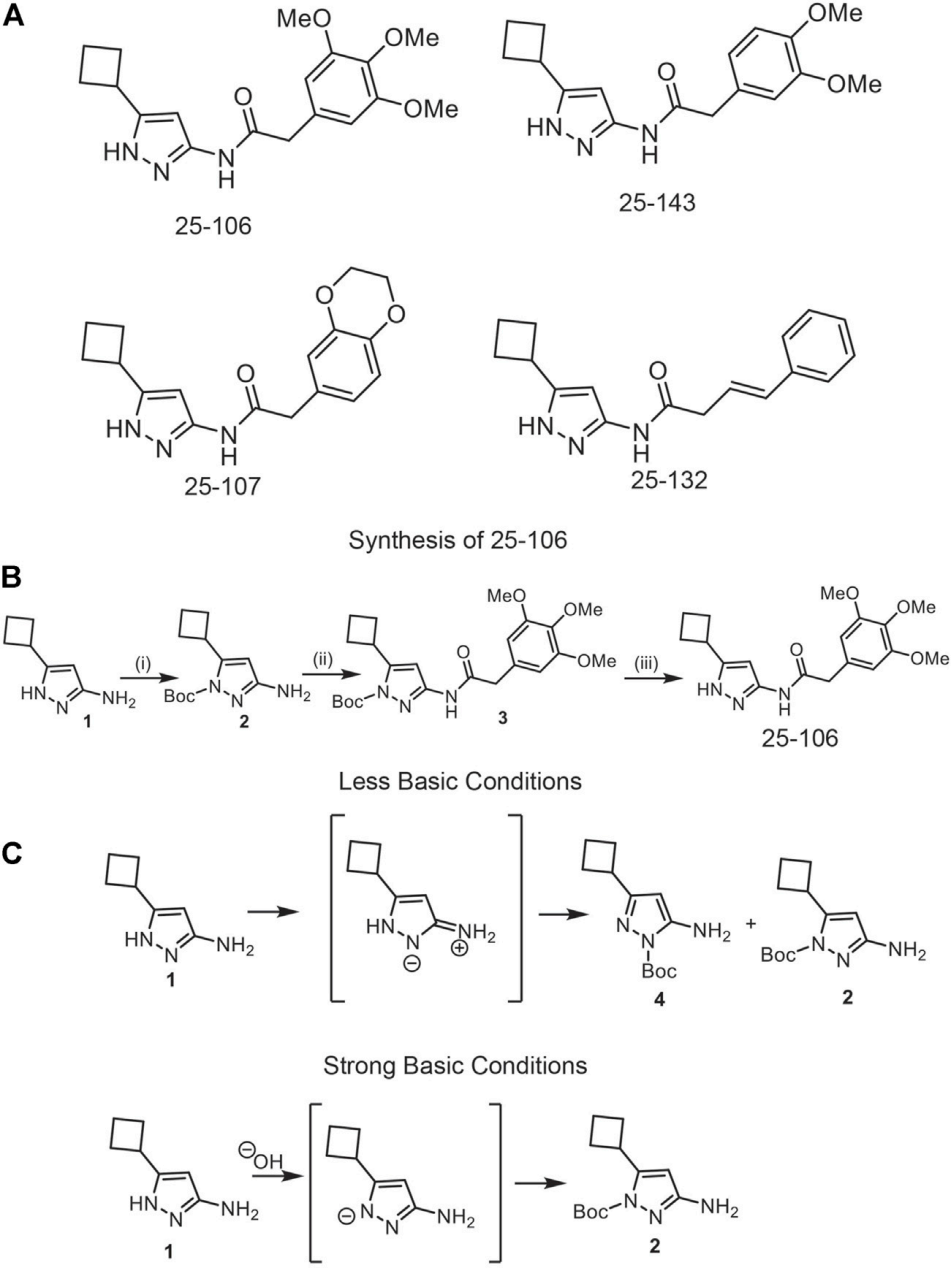
Development of new Cdk5 inhibitors. **(A)** Structures of four potential Cdk5 inhibitors: 25–106, 25–143, 25–107, and 25–132 **(B)**. Synthesis of novel inhibitor, 25–106 **(C)**. Regio-isomers are synthesized under strong or less basic conditions.

The physicochemical properties of five FDA-approved CNS drugs (droperidol, buspirone, benperidol, amisulpride, and alfentanil) (Table 1) (Ghose et al., 2012), were established as benchmarks for the query of aminopyrazole-based Cdk inhibitors with similar properties from an in-house combinatorial library. This led to the identification of three analogs, 25–143, 25–107, and 25–132, that were previously reported as Cdk2/5 inhibitors (Figure 1A) (Rana et al., 2018). In addition, we designed a fourth analog, 25–106, with an improved predicted brain/blood partition coefficient (QPlogBB), as compared to the reported compounds (Table 1). This trimethoxy aminopyrazole analog was synthesized in three steps from the commercially available starting structure, 3-amino-5-cyclobutyl-1H-pyrazole 1 (Figure 1B). The pyrazole nitrogen (N1) of 1 was protected using Boc-anhydride under strongly basic conditions. Interestingly, Boc protection under less basic conditions produced a mixture of regioisomers (4) and (2) (Figure 1C). The Boc-protected aminopyrazole (2) was reacted with 2-(3,4,5-trimethoxyphenyl) acetyl chloride which was freshly prepared from the corresponding acid. This resulted in compound 3 in 79% yield as a colorless solid. The N-Boc was deprotected on 3 using TFA to produce the title compound, 25–106, in a 76% yield as a colorless solid. Collectively, the three previously reported compounds and the newly synthesized 25–106 were predicted to serve as potent Cdk5 inhibitors, with 25–106 exhibiting the highest predicted brain/blood partition coefficient.

**TABLE 1:** Physicochemical properties of 25–132, 25–143, 25–107, 25–106, and five approved CNS drugs.^a^

Compounds	Mol. Wt	SASA^b^	Volume^c^	QPlogPo/w^d^	QPlogS^e^	QPlogBB^f^	PSA^g^	^a^N and O^h^
Droperidol	379.433	678.611	1,203.393	3.5	−4.588	−0.501	76.835	5
Buspirone	385.508	739.384	1,314.466	3.15	−4.268	−0.46	84.278	7
Benperidol	381.449	707.406	1,239.434	3.668	−5.098	−0.476	76.703	5
Amisulpride	369.478	676.461	1,224.803	1.594	−2.804	−0.88	105.738	7
Alfentanil	416.522	721.976	1,350.224	2.155	−1.611	−0.702	96.917	9
25–132	281.357	615.839	1,024.856	3.656	−5.404	−0.714	66.397	4
25–143	315.371	629.363	1,071.982	3.264	−5.032	−0.686	81.561	6
25–107	313.355	599.968	1,021.987	2.968	−4.855	−0.584	83.464	6
25–106	345.397	666.318	1,154.904	3.436	−5.273	−0.754	89.119	7

a Calculated using *QikProp*; Schrödinger.

b Total solvent accessible surface area (SASA) in square angstroms using a probe with a 1.4 Å radius.

c Total solvent-accessible volume in cubic angstroms using a probe with a 1.4 Å radius.

d Octanol−water logP (QP).

e Predicted aqueous solubility in mol dm^−3^.

f Predicted brain/blood partition coefficient.

g Van der Waals surface area of polar nitrogen and oxygen atoms.

h Number of nitrogen and oxygen atoms.

### *Ex Vivo* Screening of Cyclin-Dependent Kinase 5 Inhibitors

**FIGURE 2:**
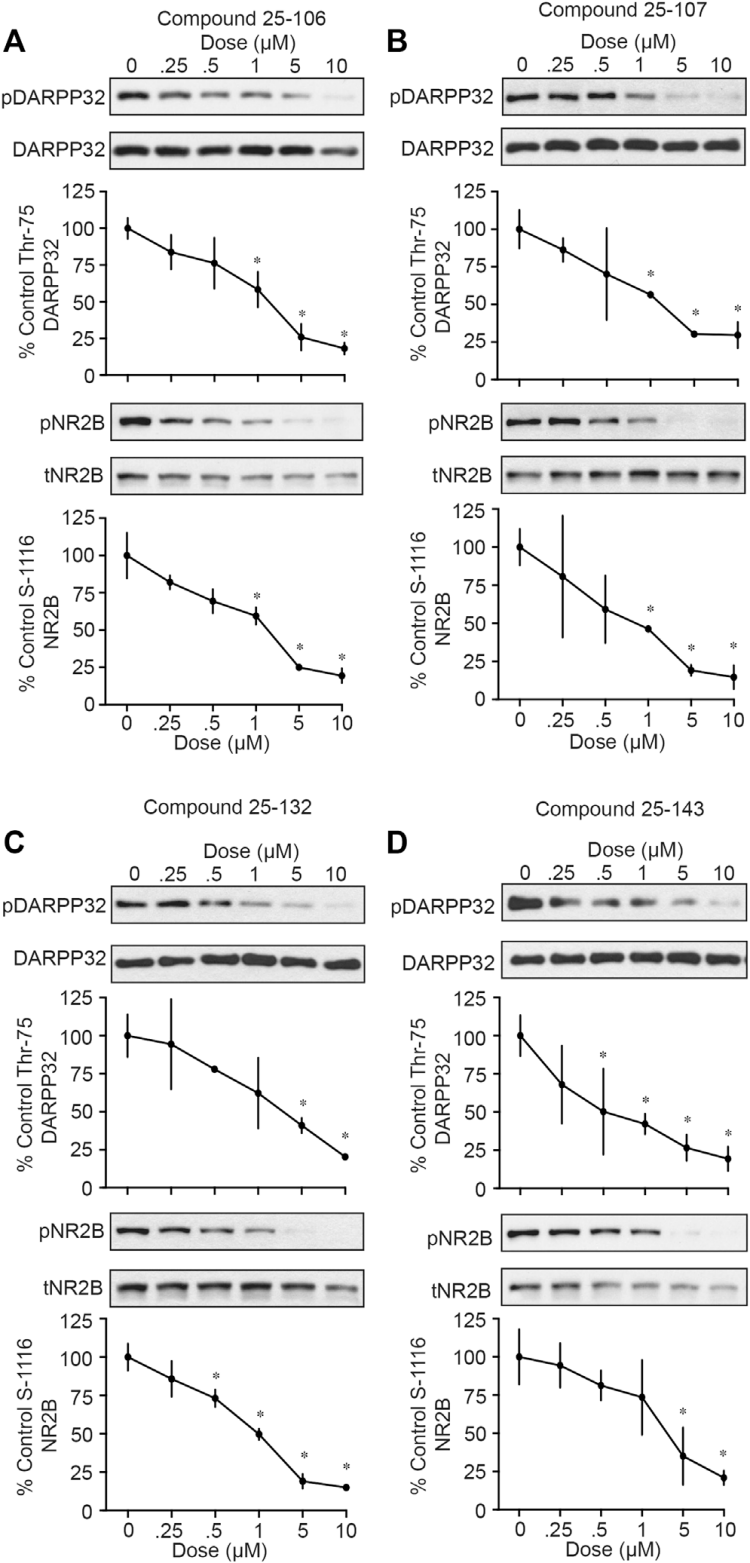
*In vitro* testing of Cdk5 inhibitors. Western blot for phosphor-DARPP32 (T75) and NR2B (S1116) of acute striatal brain slices treated (1 h) with indicated concentrations of **(A)** 25–106, **(B)** 25–107, **(C)** 25–132, and **(D)** 25–143 (*n* = four to six slices per treatment) *p <0.05 Student’s *t*-test.

In order to assess the functional inhibition of Cdk5 in intact brain tissue by the four compounds summarized in Figure 1, acutely prepared striatal slices were treated in a dose-dependent manner (0, 0.25, 0.5, 1, 5, and 10 µM) with each inhibitor for 1 h, and Cdk5 activity was assessed *via* immunoblotting the phosphorylation of established Cdk5 sites, phospho-Thr75 DARPP-32 and phospho-Ser1116 NR2B (Bibb et al., 1999; Plattner et al., 2014). All four compounds tested, 25–106 (Figure 2A), 25–107 (Figure 2B), 25–132 (Figure 2C) and 25–143 (Figure 2D), displayed comparable efficacy as *ex vivo* inhibitors of Cdk5-dependent phosphorylation. All compounds caused significant reductions in Cdk5-dependent phosphorylation of DARPP-32 and NR2B with IC_50_ values ranging approximately 0.5–1 µm. Thus, each of these newly synthesized compounds functioned in intact brain tissue as potent Cdk5 inhibitors and thus exhibited relatively equal potential to serve as anti-Cdk5 therapeutics.

### Specificity and Efficacy of 25–106

**FIGURE 3:**
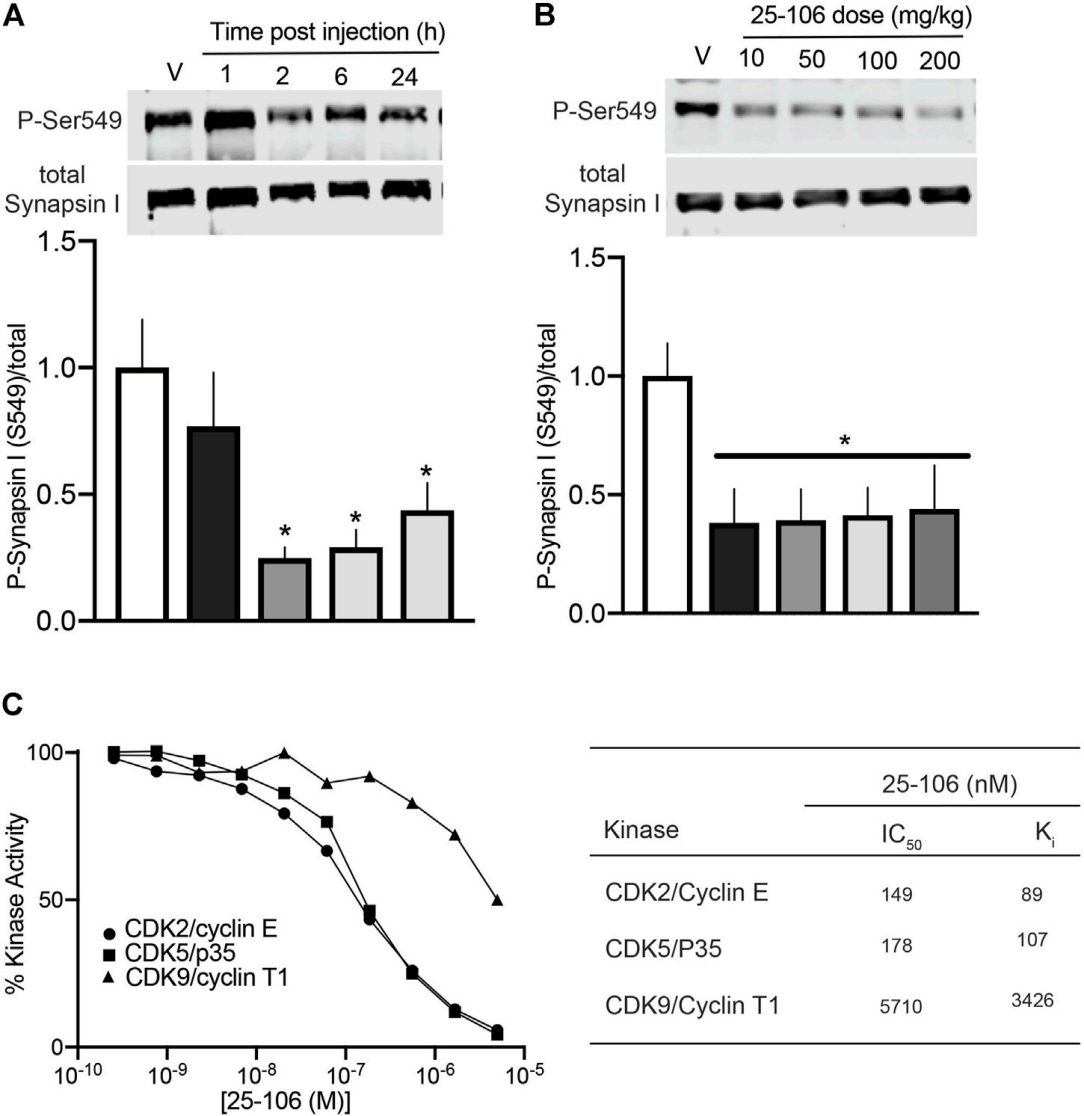
*In vivo* efficacy and *in vitro* specificity of 25–106 **(A)**. Quantitative immunoblot analysis of striatal brain lysates for phospho-Ser549 synapsin I after dosing 50 mg/kg 25–106 across the times indicated (*n* = 4 per group) **(B)**. Quantitative immunoblot of phospho-Ser549 synapsin I 24 h post administration across doses indicated (*n* = 4 per group). **(C)**. *In vitro* kinase assay (30 min) with Cdk2/Cyclin E, Cdk5/p25, and Cdk9/Cyclin T1 treated with 0–5 µM 25–106 (left) calculated IC_50,_ K_i_ values of each kinase (right). *p <0.05 student’s *t*-test.

Each of the four compounds tested in brain slices was further screened for their ability to penetrate the blood–brain barrier (BBB) and inhibit Cdk5-dependent activity *in vivo.* For these studies, cohorts of mice were treated with each compound *via* I.V. injection (50 mg/kg), and brain striatal lysates from various time points were subjected to quantitative immunoblot analysis for phosho-Ser549 Synapsin I levels. Three compounds, 20–107, 20–132, and 20–143, had no effect on Cdk5 activity compared to that of vehicle alone (data not shown). In contrast, 25–106 significantly reduced phospho-Ser549 synapsin I levels in the striatum 2 h after I.V. injection (Figure 3A). Moreover, this effect persisted even 24 h after dosing. Interestingly, a dose–response test revealed that 25–106 caused significant reduction in striatal phospho-Ser549 synapsin I levels 24 h post injection across a 20-fold dose range, with approximately equal efficacy at 10, 50, 100, and 200 mg/kg.

For assessment of the *in vitro* selectivity of 25–106, we conducted a dose–response kinase inhibition assay with three highly homologous Cdks, Cdk2/CyclinE, Cdk5/p25, and Cdk9/CyclinT1 (Sonawane et al., 2016). 25–106 exhibited dose-dependent inhibition of all three kinases tested (Figure 3C). However, the compound showed greater than 30-fold selectivity for Cdk2 and Cdk5 than Cdk9.

### Molecular Modeling of Cyclin-Dependent Kinase 5 Inhibitors

**FIGURE 4:**
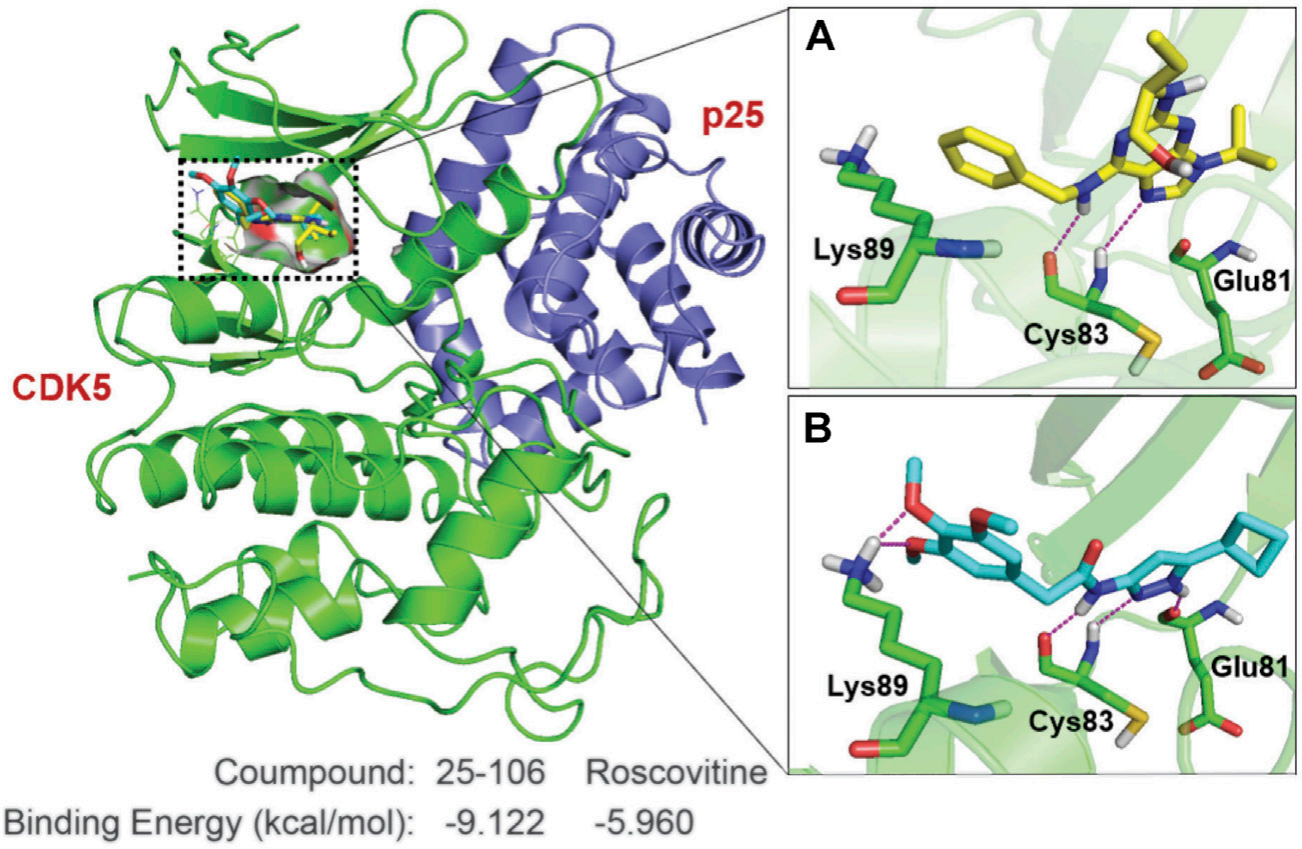
Key interactions made by 25–106 and roscovitine with CDK5/p25. CDK5 (green cartoon)/p25 (blue cartoon) (PDB:1H4L). Hydrogen bonds are shown in magenta lines. **(A)** Roscovitine (represented by yellow sticks), **(B)** 25–106 (represented by cyan sticks).

Due to the prolonged inhibition of Cdk5 by 25–106 observed in (Figure 3), we decided to investigate the specific drug interactions of 25–106 with Cdk5 as compared to the well-established Cdk5 inhibitor, roscovitine. The crystal structure of a complex between CDK5 and p25 was used to elucidate the binding mode of 25–106 and compare it to that of roscovitine (Tarricone et al., 2001). A binding pocket analysis was performed on the CDK5/p25 protein structure (PDB: 1H4L) using the SiteMap tool by Schrödinger Inc. (Halgren, 2009). The ATP binding pocket yielded site and druggability scores of 1.05 and 1.07, respectively, and was selected for docking. The analysis was performed using Glide XP with 25–106 and roscovitine, which revealed that the compounds had similar binding modes with CDK5. The hydrogen bonds formed by the purine core in roscovitine with the hinge region residue Cys^83^ are mimicked by the aminopyrazole core in 25–106. The cyclobutyl group on the pyrazole ring of 25–106 and the isopropyl group on the imidazole ring of roscovitine occupy the same hydrophobic binding pocket (Figure 4). In addition to the common hydrogen bond interactions with the hinge region residue Cys^83^, 25–106 forms hydrogen bonds with Glu^81^ and Lys^89^ (Figure 4). The extra bonds formed by 25–106 result in a lower predicted required binding energy of −9.122 kcal/mol than −5.960 kcal/mol for roscovitine (Figure 4).

### Detection of 25–106 in Preclinical Biospecimens

**FIGURE 5:**
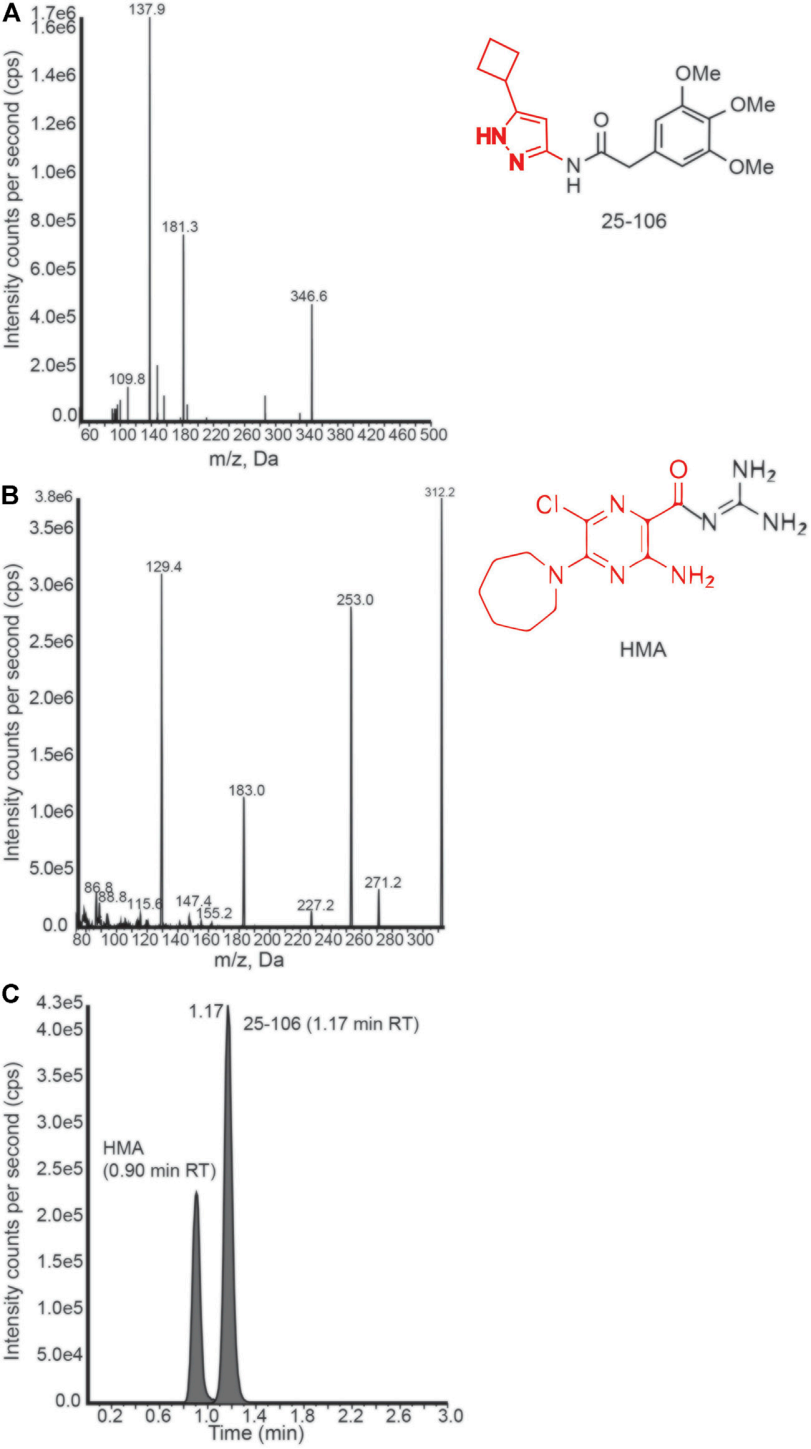
LC-MS/MS detection of 25–106 **(A)**. Mass spectrum peaks generated from ion fragmentation of parent compound 25–106 (right). Major peak (137.9 highlighted in red). **(B)** Mass spectrum peaks generated from the ion fragmentation internal standard (IS) HMA (right). Major peak (129.4 outlined red). **(C)** Chromatogram of 25–106 (125 ng) and HMA (100 ng) mixture displaying two temporally distinct peaks.

In order to derive the pharmacokinetic properties of 25–106 *in vivo*, we developed a protein precipitation and high-performance liquid chromatography with tandem mass spectrometry (LC-MS/MS) detection method to assess the distribution, concentration, and elimination of 25–106 throughout several organ systems. For these studies, 5-(N,N-hexamethylene) amiloride (HMA) was used as an internal standard. Mass spectra product transitions were observed as 25–106 displayed a prominent peak of the parent compound at 346.6 m/z, with the product transition for 25–106 displaying a larger peak at 137.9 m/z (Figure 5A). Likewise, the internal standard HMA displayed a parent peak of 312.2 m/z with subsequent fragmentation to 129.4 m/z (Figure 5B). Chromatograms displaying the selectivity for each compound’s transition state show distinctly detected peaks for a 100 ng/ml concentration of HMA (0.90 min) and a 125 ng/ml concentration of the extracted calibration standard of 25–106 (1.17 min) (Figure 5C). Altogether, these data display a selective approach in detecting and measuring the concentration of 25–106 that can be applied to various tissue samples.

### Pharmacokinetic Profile of 25–106 in Mice

**FIGURE 6:**
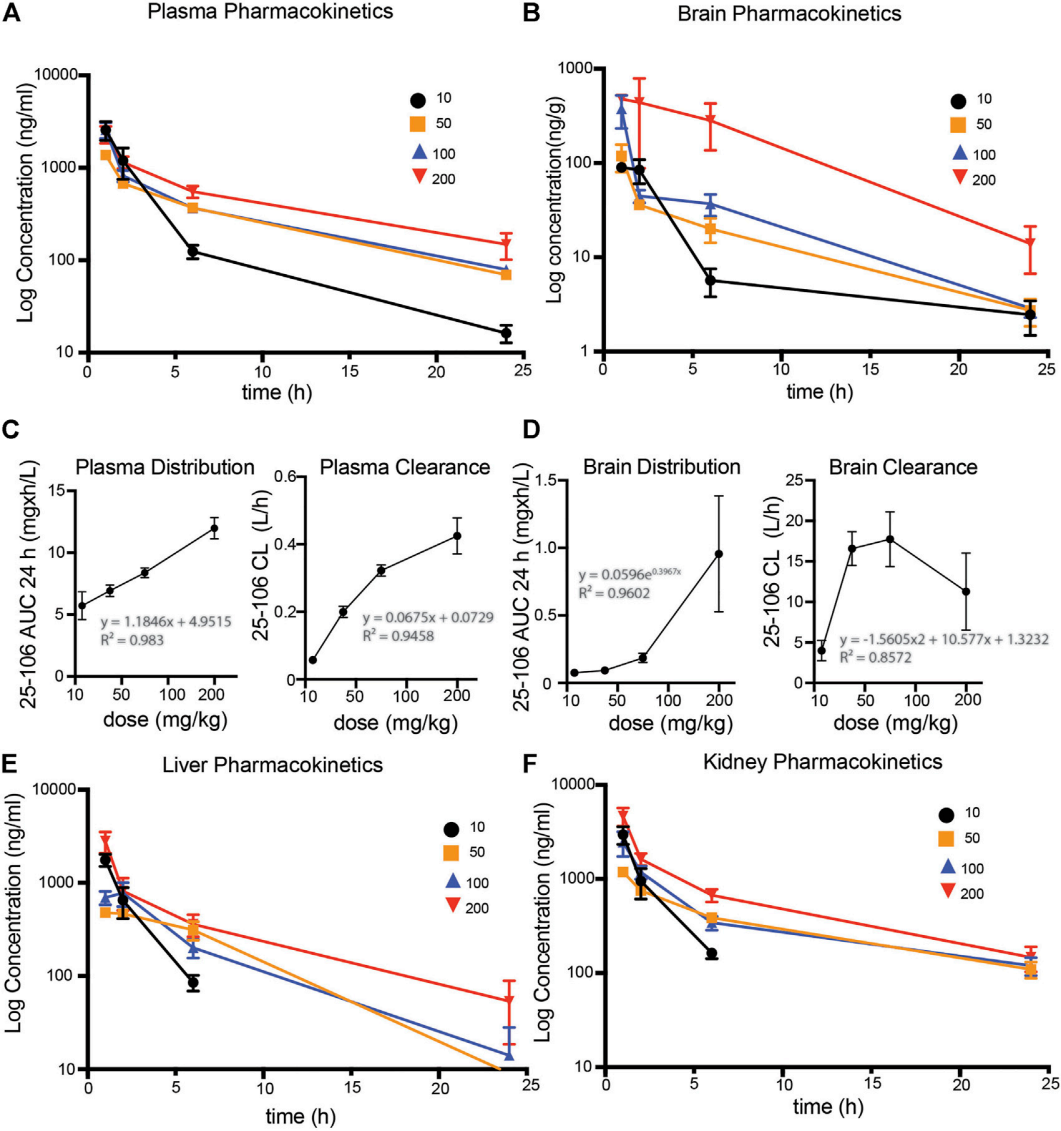
Pharmacokinetic profile of 25–106 **(A,B)**. LC-MS/MS detected 25–106 used at four doses (10, 50, 100, and 200 mg/kg) across 1, 2, 6, and 24 h post injection in the plasma **(A)** and brain **(B)**. **(C)** distribution and clearance of 25–106 in plasma. **(D)** Distribution and clearance of 25–106 in the brain. **(E,F)**. LC-MS/MS detected a concentration of 25–106 in the liver **(E)** and kidney **(F)** (*n* = 4 for each treatment).

To assess the pharmacokinetic and pharmacodynamic parameters of 25–106 *in vivo*, we utilized the LC-MS/MS detection method established above (see Figure 5). The mice were administered IV injections of 25–106 doses of 10, 50, 100, or 200 mg/kg. The animals were euthanized, and organs were harvested at 1, 2, 6, and 24 h post injection. Within the same experiment, tissues were used for biochemical analysis of 25–106 effects on Cdk5 activity (see Figures 3A, B). 25–106 showed rapid distribution following 1 h post injection in both the plasma and brain at all four doses tested (Figures 6A,B). 25–106 concentrations steadily decreased over time, remaining detectable in the brain and plasma 24 h after injection. From the concentration-time curves derived for the plasma and brain, several pharmacokinetic parameters, including maximum concentration (C_max_), elimination rate constant (K_e_), half-life (T_1/2_), last measurable concentration (C_last_), 24-h area-under-curve (AUC_24h_), distribution volume (V), and clearance (CL), were calculated (Tables 2, 3). 25–106 showed rapid uptake in the plasma across all doses tested (10, 50, 100, 200 mg/kg), reaching average plasma C_Max_ values of 2567, 1375, 2,577, and 2,322 ng/ml, respectively (Table 2). In the plasma, 25–106 demonstrated linearity between the AUC vs. dose (*R* ^2^ = 0.983) (Figure 6C). The CL of 25–106 from the plasma displayed varied but linear results as well (*R* ^2^ = 0.9454) (Figure 6C). Of note, the half-life T_1/2_ of 25–106 in the plasma appeared to increase with each increasing dose injected (Table 2).

**TABLE 2:** Pharmacokinetic values of 25–106 in plasma.

Dose (mg/kg)	Ke (1/h)	T_1/2_ (h)	C_max_ Ng/ml	C_last_ Ng/ml	AUC_24h_ h*mg/L	V L	CL L/h
10	0.11 ± 0.03	6.50 ± 2.36	2,567.50 ± 1,179.47	16.26 ± 7.00	5.71 ± 2.26	0.49 ± 0.08	0.06 ± 0.02
50	0.09 ± 0.01	7.48 ± 0.41	1,375.00 ± 184.30	69.68 ± 16.17	6.93 ± 0.93	2.14 ± 0.34	0.20 ± 0.03
100	0.09 ± 0.02	8.37 ± 1.77	2,577.50 ± 1,004.73	79.18 ± 16.03	8.38 ± 0.78	3.85 ± 0.55	0.32 ± 0.03
200	0.08 ± 0.04	11.04 ± 6.38	2,322.50 ± 972.71	148.65 ± 94.09	11.98 ± 1.74	6.12 ± 2.08	0.43 ± 0.11

Values expressed represent group averages ± SD, single injection (*n* = 4 per group).

**TABLE 3:** Pharmacokinetic values of 25–106 in the brain.

Dose (mg/kg)	Ke (1/h)	T1/2 (h)	Cmax Ng/g	Clast Ng/ml	AUC_24h_ h*mg/L	V L	CL L/h
10	0.06 ± 0.06	17.20 ± 10.39	101.13 ± 23.65	2.27 ± 1.44	0.08 ± 0.02	77.39 ± 25.63	3.98 ± 2.18
50	0.11 ± 0.02	6.23 ± 1.12	118.25 ± 76.70	2.73 ± 1.75	0.09 ± 0.03	151.24 ± 58.36	16.57 ± 4.19
100	0.14 ± 0.02	5.03 ± 0.69	376.88 ± 289.08	2.88 ± 1.16	0.18 ± 0.07	126.99 ± 46.68	17.75 ± 6.77
200	0.17 ± 0.04	4.39 ± 1.19	668.75 ± 315.86	13.97 ± 14.54	0.96 ± 0.86	76.07 ± 73.66	11.29 ± 9.51

Values expressed represent group averages ± SD, single injection (*n* = 4 per group).

Similar to the plasma, the highest distribution of 25–106 in the brain was observed 1 h post injection and decreased over time, remaining detectable 24 h after injection. Unlike the plasma, 25–106 distribution in the brain increased exponentially with the dose injected (AUC vs. dose), reaching C_max_ results of 101.13, 118.25, 376.88, and 668.75 ng/g for 10, 50, 100, and 200 mg/kg injections, respectively (Figure 6D; Table 3). The T_1/2_ of 25–106 in the brain remained relatively constant (excluding the lowest dose of 10 mg/kg), with an average half-life of 6.23, 5.03, and 4.39 h for 50, 100, and 200 mg/kg injections, respectively (Table 3). Interestingly, the clearance (CL) of 25–106 from the brain stabilized and decreased at the highest doses tested, with the average CL of 10 mg/kg (3.98 L/h), 50 mg/kg (16.5 L/h), 100 mg/kg (17.7 L/h) and 200 mg/kg (11.29 L/h) (Figure 6D; Table 3). Taken together, these data demonstrate that 25–106 show the properties of a linear drug in the plasma which enters the brain in a dose-dependent exponential manner.

In order to see if there was a 25–106 distribution in other off-target organ systems, in the same experiment, we again utilized the LC-MS/MS detection of liver and kidney samples. Much like the plasma and brain, 25–106 showed dose-dependent increased concentrations in the liver (Figure 6E) and kidney (Figure 6F), with the highest concentrations detected at the 1 h post-injection time point. In the liver, across all doses tested, 25–106 remained undetectable within some samples. Therefore, only the C_max_, C_last_, and AUC could be determined for these samples (Table 4). Within the kidney, 25–106 was only undetectable at the lowest dose (10 mg/kg). For each other dose, all values of C_max_, K_e_,T_1/2_, C_last_, AUC, V, and CL were calculated (Table 5). Taken together, these results demonstrate 25–106’s temporal and dose-related abilities to distribute into several organ systems, including the brain.

**TABLE 4:** Pharmacokinetic values of 25–106 in the liver.

Dose (mg/kg)	Ke (1/h)	T_1/2_ (h)	C_max_ Ng/ml	C_last_ Ng/ml	AUC_24h_ h*mg/L	V L	CLL/h
10	-	-	1760 ± 526.43	85.18 ± 32.60	3.08 ± 1.01	-	-
50	-	-	512.75 ± 22.01	252.73 ± 200.06	2.76 ± 0.98	-	-
100	-	-	867.5 ± 378.22	150.9 ± 103.92	3.32 ± 1.28	-	-
200	-	-	2,807.5 ± 1,436.49	310.9 ± 247.48	5.44 ± 1.95	-	-

Values expressed represent group averages ±SD, single injection (*n* = 4 per group).

-Not measurable within the constraints of PK study as 25–106 was undetectable in samples at 24 h.

**TABLE 5:** Pharmacokinetic values of 25–106 in the kidney.

Dose (mg/kg)	Ke (1/h)	T_1/2_ (h)	C_max_ Ng/ml	C_last_ Ng/ml	AUC_24h_ h*mg/L	V L	CL L/h
10	-	-	2,952.5 ± 1,259.90	163 ± 41.67	6.30 ± 1.69	-	-
50	0.07 ± 0.024	10.23 ± 2.99	1,185.5 ± 249.96	109.33 ± 42.90	7.64 ± 1.28	78.88 ± 16.88	5.55 ± 1.25
100	0.06 ± 0.02	13.05 ± 4.56	2,580 ± 1,272.50	119.88 ± 51.83	9.42 ± 3.06	172.86 ± 89.05	9.25 ± 2.79
200	0.09 ± 0.049	9.65 ± 5.34	4,657.5 ± 2048.58	146.45 ± 87.11	14.43 ± 4.63	163.98 ± 55.79	13.61 ± 6.82

Values expressed represent group averages ± SD, single injection (*n* = 4 per group).

-Not measurable within the constraints of PK study as 25–106 was undetectable in samples at 24 h.

### Neurobehavioral Modulation by 25–106

**FIGURE 7:**
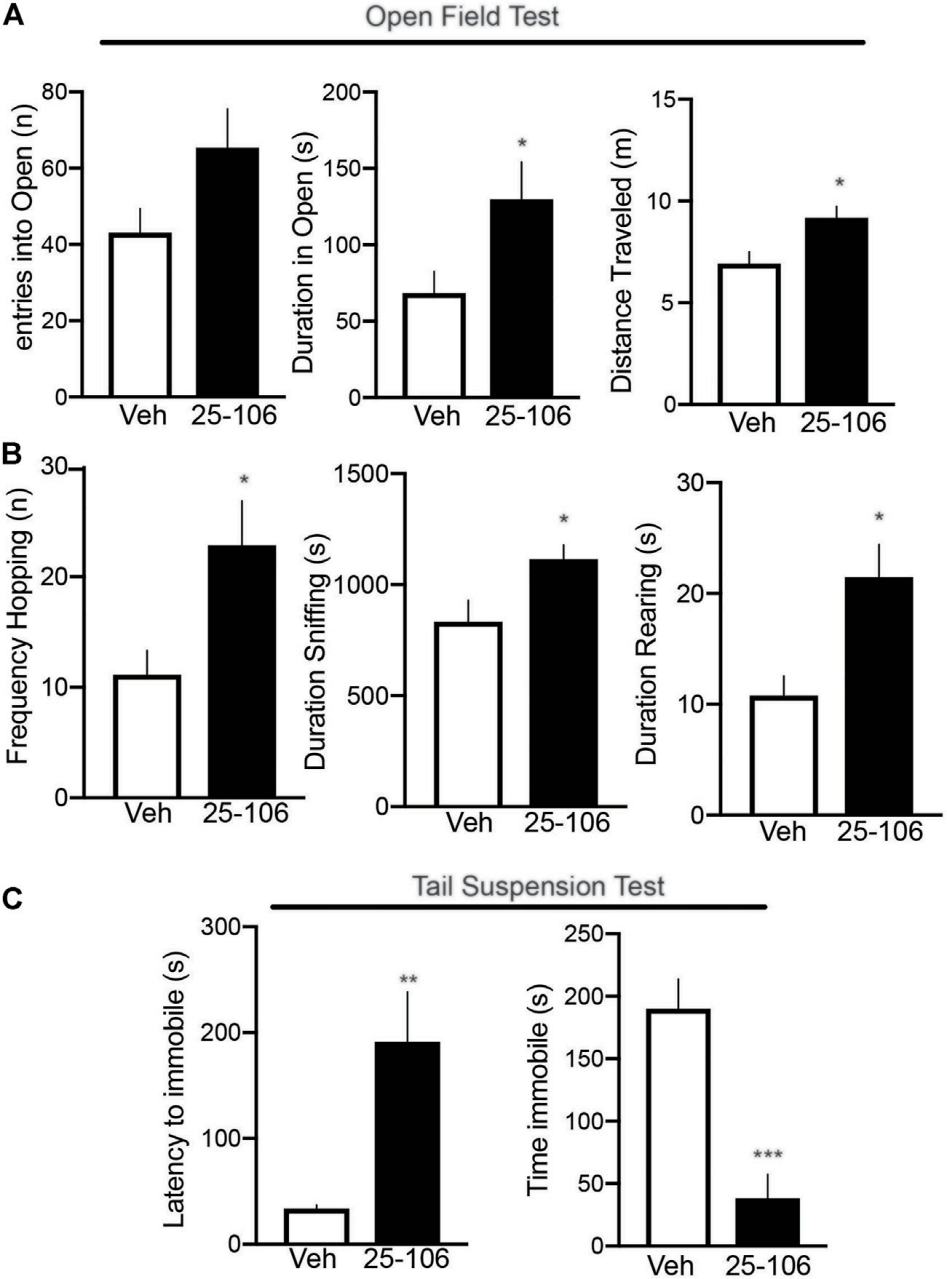
Neurobehavioral effects of systemic Cdk5 inhibition. **(A)** Open-field testing (*n* = 10–12 per group) in mice treated with vehicle or (50 mg/kg) 25–106 1 h after injection. Parameters displayed include entries into the open, duration in the open, and distance traveled. **(B)** Stereotypic behaviors were assessed during the open-field testing. **(C)** Effect of systemic 25–106 (50 mg/kg) treatment vs. vehicle on behavioral responses in the tail suspension test (*n* = 11 per group) in vehicle or 25–106–treated mice 6 h s after injection. *p <0.05 Student’s *t*-test, **p <0.01 student’s *t*-test, and ***p <0.001 student’s *t*-test.

To establish a functional effect of pharmacological Cdk5 inhibition in the brain, we tested mouse performance in two behavioral paradigms previously shown to be altered in Cdk5 knockout mice: the open-field maze and the tail suspension test (Plattner et al., 2015). The open-field maze was used to measure locomotion and anxiety-like behavior (Cacace et al., 2011; Seibenhener and Wooten, 2015). Mice were injected with either vehicle or 50 mg/kg of 25–106 and allowed to freely explore an open-field maze for 1 h with locomotor activity being tracked (Figure 7). The mice injected with 25–106 displayed an increased number of entries into the open portion of the maze and a longer duration of time spent in the open portion of the maze as compared to vehicle-injected mice (Figure 7A). In addition, mice injected with 25–106 displayed increased hopping bouts, increased time sniffing, and increased rearing behaviors (Figure 7B).

Next, we tested the effects of 25–106 on mouse performance in the tail-suspension test to measure depressive-like phenotypes in mice (Cryan et al., 2005; Can et al., 2012). Mice treated with 50 mg/kg 25–106 displayed an increased latency to suspend immobility and displayed a reduction in total immobility time as compared to vehicle-injected mice (Figure 7C). Thus, 25–106 modulated several neurobehavioral behaviors that have previously been linked to Cdk5.

## Discussion

A multitude of studies have implicated Cdk5 as an important neuronal signal transduction mediator in a variety of diseases and suggest anti-Cdk5 approaches as rational therapeutic strategies (Lee et al., 2000; Hung et al., 2005; Camins et al., 2006; Alvira et al., 2008; Giese, 2014). Initial enthusiasm for anti-Cdk5 treatment for neurodegenerative diseases such as Alzheimer’s disease (AD) led to the generation of a number of lead compounds which showed reasonable potency *in vitro*. However, the lack of specificity, recognition of potential postmortem confounds in human AD studies, and possibility that chronic inhibition of Cdk5 might lead to neuronal hyperexcitability and epileptiform seizures, as found in aged Cdk5 conditional knockout mice, all contributed to impedance in the pursuit of anti-Cdk5 therapeutics, leaving first- and second-generation compounds, which had poor brain permeable systemic properties, unsuitable for *in vivo* study (Yoo and Lubec, 2001; Hawasli et al., 2009). For example, pharmacokinetic analysis has demonstrated that roscovitine could distribute into the brain. However, a rapid half-life with no detectable active metabolites was limiting (Vita et al., 2005).

Here, we show 25–106 is a nonselective Cdk2/5 inhibitor that displays low nM IC_50_ and K_i_ values. While the shared specificity for these two kinases is consistent with the *in vitro* profiles for other Cdk5 inhibitors, it should be noted that very low levels of Cdk2 are detected in the brain (Bibb et al., 2001b). Moreover, aminopyrazole-based inhibitors selectively inhibit Cdk5 substrates over Cdk2 substrates in cellular models (Robb et al., 2018). CDK inhibitors, in general, lack selectivity for individual CDKs, given the strong homology within the catalytic domains across kinase family members. While this lack of specificity is most frequently evidenced by *in vitro* phosphorylation effects, where the drugs can act at their highest potency on purified and often recombinant kinases, within the intracellular milieu, conditions and interactions with other proteins or cell constituents may afford different or even more selective inhibition profiles (Knight and Shokat, 2005; Smyth and Collins, 2009). Indeed, other Cdk5 inhibitors in this class of molecules have been shown to be more selective for Cdk5 than Cdk1/2 in cultured cells (Pozo et al., 2013; Gupta et al., 2022). However, any off-target or toxic effects of systemic inhibition of Cdk2 by 25–106 remain unknown.

Although it has been postulated that pilot inhibitors of Cdk5, such as enantiomers of roscovitine, display improved BBB permeability and functional protection following ischemia, Cdk5 site-directed decreases in phosphorylation stoichiometry have not been clearly shown (Menn et al., 2010). Here, we introduce a new Cdk5 inhibitor and robustly characterize its pharmacokinetic and dynamic responses within several organ systems, including the brain. We observed the highest concentration of 25–106 in the brain at the earliest time point measured (1 h post injection). Therefore, peak levels in the brain are likely achieved rapidly after injection. In order to ascertain the true T_max_ of this molecule, future studies of 25–106’s brain distribution across the initial post-injection period will be necessary. Interestingly, although the highest concentrations of 25–106 detected in the brain were at the 1 h post-injection timepoint, Cdk5-dependent phosphorylation remained unchanged 1 h post injection. This likely indicates a time-dependent delay for 25–106 to diffuse into neurons and appropriately bind and inhibit Cdk5-dependent phosphorylation states. No demethylated metabolite structures of 25–106 were detected. The degradation of 25–106 into active or inactive metabolites and their subsequent distribution within the body remain unknown. 25–106 remained detectable in the brain 24 h after injection but was also well-absorbed by peripheral tissues such as the liver and kidney, which could contribute to off-target effects or toxicity arising from prolonged treatment. Therefore, the development of derivatives of 25–106 with greater brain permeability and tissue specificity is a reasonable goal. Molecular modeling of 25–106 as compared to another Cdk5 inhibitor, roscovitine, revealed that 25–106 forms two additional bonds within the hinge region of the ATP binding domain of Cdk5. These extra binding motifs may explain the high activity of 25–106, the decreased binding energy required as compared to Roscovitine, and the prolonged inhibition of Cdk5-dependent substrates.

Previous studies have demonstrated striatal Cdk5 knockout or inhibition results in reduced anxiety-like behaviors in mice *via* regulation of phosphodiesterase4 (PDE4) activity and protein kinase A (PKA) signaling (Plattner et al., 2015). Impaired PKA activity is associated with major depressive disorder and inhibition of PDE4 and elevated levels of cAMP induce antidepressant effects (Fujita et al., 2012; O’donnell and Zhang, 2004; O’donnell and Xu, 2012). Here, we observed similar behavioral changes in two paradigms with brain-wide pharmacological inhibition of Cdk5. While 25–106 may impart the same mechanistic regulation of PKA activity, this mechanism is not explored here. Other studies have implicated Cdk5 activity in the septum in regulating anxiety-like behaviors in animals (Bignante et al., 2008). Therefore, the exact mechanisms by which 25–106 mediates the phenotypes observed here remain unknown. Alternatively, chronic deletion of Cdk5 in aged mice results in increased acoustic startle responses, a well-defined behavioral paradigm to measure animal responses to an emotional context or stressor (Hawasli et al., 2009; Hantsoo et al., 2018; Pantoni et al., 2020). Since this behavioral adaptation is only observed in aged mice with chronic depletion of Cdk5, we did not assess this paradigm using acute pharmacological inhibition of Cdk5. Chronic treatment and subsequent behavioral changes remain an interesting area of exploration. Additional studies have demonstrated that Cdk5’s cofactor p35 increases following stress exposure and Cdk5 activity increases following restraint stress (Bignante et al., 2010; Papadopoulou et al., 2015). In addition, the reduced anxiety-like behaviors observed here may also be in part due to regulation of various neuropeptides that modulate stress and anxiety-like behaviors (Kormos and Gaszner, 2013; Rana et al., 2022). Cdk5 activity has been shown to increase in mice following corticosterone injections, and this increase in activity correlates with increased phosphorylation of the glucocorticoid receptor following acute stress. However, direct phosphorylation by Cdk5 was not observed. Cdk5’s role in regulation of these neuropeptides implicated in stress remains an understudied area (Papadopoulou et al., 2015).

As perhaps the first robust systemic inhibitor, 25–106 represents an exciting and expandable and translatable pharmacological tool to study the function of Cdk5 activity in wild-type animals. Achieving systemic applicability may be considered a step forward toward the testing of Cdk5 inhibitors to treat neuropsychiatric and neurodegenerative diseases. This provides a promising landscape for future studies to assess the effects of brain-permeable Cdk5 inhibitors to combat stress, anxiety, depression, addiction, cancer, and neurodegeneration.

## Data Availability

The raw data supporting the conclusion of this article will be made available by the authors; without undue reservation.
